# Genetic and linguistic non-correspondence suggests evidence for collective social climbing in the Kol tribe of South Asia

**DOI:** 10.1038/s41598-020-61941-z

**Published:** 2020-03-27

**Authors:** Anshika Srivastava, Prajjval Pratap Singh, Audditiya Bandopadhyay, Pooja Singh, Debashruti Das, Rakesh Tamang, Akhilesh Kumar Chaubey, Pankaj Shrivastava, George van Driem, Gyaneshwer Chaubey

**Affiliations:** 1 Cytogenetics Laboratory, Department of Zoology, Banaras Hindu University, Varanasi, 221005 India; 2 Department of Zoology, University of Calcutta, Kolkata, 700019 India; 3 Krishi Vigyan Kendra, Singrauli, Jawaharlal Nehru Krishi Vishwavidyalay, Jabalpur, Madhya Pradesh 462038 India; 4 DNA Fingerprinting Unit, State Forensic Science Laboratory, Department of Home (Police), Government of MP, Sagar, 470001 India; 5 Institut für Sprachwissenschaft, Universität Bern, 3012 Bern, Switzerland; 6 Sydney Social Sciences and Humanities Advanced Research Centre, University of Sydney, Sydney, Australia; 7 Estonian Biocentre, Institute of Genomics, University of Tartu, Tartu, 51010 Estonia

**Keywords:** Biological anthropology, Structural variation

## Abstract

Both classical and recent genetic studies have unanimously concluded that the genetic landscape of South Asia is unique. At long distances the ‘isolation-by-distance’ model appears to correspond well with the genetic data, whereas at short distances several other factors, including the caste, have been shown to be strong determinant factors. In addition with these, tribal populations speaking various languages add yet another layer of genetic complexity. The Kol are the third most populous tribal population in India, comprising communities speaking Austroasiatic languages of the Northern Munda branch. Yet, the Kol have not hitherto undergone in-depth genetic analysis. In the present study, we have analysed two Kol groups of central and western India for hundreds thousands of autosomal and several mitochondrial DNA makers to infer their fine genetic structure and affinities to other Eurasian populations. In contrast, with their known linguistic affinity, the Kol share their more recent common ancestry with the Indo-European and Dravidian speaking populations. The geographic-genetic neighbour tests at both the temporal and spatial levels have suggested some degree of excess allele sharing of Kol1 with Kol2, thereby indicating their common stock. Our extensive analysis on the Kol ethnic group shows South Asia to be a living genetics lab, where real-time tests can be performed on existing hypotheses.

## Introduction

**Figure 1:**
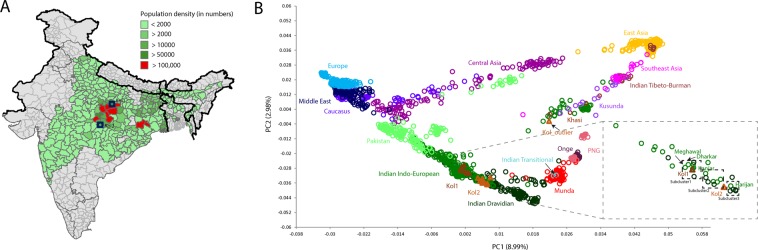
(**A**) The geographic distribution of Kol population and our sampling locations. (**B)** The principal component analysis (PCA) of Eurasian populations showing the placement of Kol alongwith the South Asian cline. The subplot shown on the right side is plotted by using mean values of the populations.

The Indian subcontinent is renowned for the cultural, linguistic and genetic diversity of its inhabitants^1,2^. This diversity has mainly arisen, in part, through long term human settlement, social customs and genetic drift^3–5^. Broadly, Indian populations can be categorised as the castes, tribes, linguistic and religious communities. Presently, India counts hundreds of tribal groups, belonging to four major language families; Austroasiatic, Dravidian, Indo-European and Tibeto-Burman^6,7^. Kol is one of them, with their major concentration in Central India (Fig. 1A). Kol is another name for Ho, whose language is a member of the Kherwarian cluster within the Northern Branch of the Munda subgroup of Austroasiatic language family^7–9^. In fact, the language family came to be known as ‘Mon-Khmer-Kolarian’ when Francis Mason first identified that Kol and the other Munda languages were related to the Mon language of eastern Burma and Thailand in 1854. He suggested that these Munda or ‘Kolarian’ languages of India and the ‘Mon-Annam’ languages of Southeast Asia, collectively belonged to one and the same language family^10^. The language family was given its current name ‘Austroasiatic’ in 1904 by Wilhelm Schmidt^11–15^.

The word Kol is derived from the Mundari word, ‘*ko’* which means ‘they and others’^16^. They are mainly concentrated in Central India and regions of Deccan plateau (Fig. 1A). Kols claim themselves to be descendants of epic *Ramayana* character Savari or Sheori, calling her “Mother of all Kols”, and also believe they once inhabited the hills of Rajasthan with another prominent tribe Bhils and helped Rana Pratap, Rajput King of Mewar Rajasthan, in his struggle with the Mughal invaders^17^.

The linguistic association of Kol is conflicting^6,11,12,16^, therefore we undertook this study to dissect a fine-grained genetic structure of them. We used large number of autosomal and mitochondrial DNA markers to investigate the incompatible association of Kols as well as their inter and intra population affinities (Supplementary Tables 1 and 2).

## Results and Discussion

Caste and tribal affinities in South Asia are factors known to have played a vital role in shaping the genetic landscape of the subcontinent^4,18,19^. In our attempt to understand this genetic complexity, we have assessed the ancestry and geneflow pattern of the major tribal populations of South Asia^7,20,21^. In present study we evaluated the genetic affinities of the Kol population, which, as the third largest tribal population of South Asia, comprises ~1.7 million people (Fig. 1A).

In conducting our genetic study, we first ascertained the classical ethnographic work, which has suggested Kol as an Austroasiatic (Munda) speaker^17^. As observed previously, the Austroasiatic speakers in India fall out of the South Asian cline due to their Southeast Asian genetic affinity^22–25^. Therefore, we expected to see their (Kol) clustering with the Munda speakers. However, in the principal component analysis (PCA), both of the studied Kol groups aligned along the South Asian cline with clusters formed by a large number of Indo-European and a few Dravidian speakers (Fig. 1B). Although, the Kols are geographically immediate neighbours of Mundari and Transitional populations, they remarkably exhibit no attraction towards Austroasiatic or Transitional populations (Fig. 1B). At the intra-population level, both of the Kol groups were distinct from each other, suggesting their long-term separation or a possibility of assimilation of different neighbouring tribal groups into a single ethnolinguistic unit called Kol. More specifically, we see three sub-clusters in the vicinity of both of the Kol groups (Fig. 1B). Kol1 and Kol2 fall in the subclusters1 and 2 respectively. Kol1 falls in the subcluster1 with Meghwal, Kurmi, Dharkars, Kanjars (Indian Indo-European), and Lambadi (Dravidian) populations, whilst Kol2 was found to be in-between subclusters 2 and 3 harbouring Dravidian (Sakilli and North Kannadi) and Indo-European (Harijan) populations (Fig. 1B). It is noteworthy, that both of the Kol groups largely share a closer genetic relationship with the majority of the Scheduled caste populations living to their north, speaking Indo-European languages.

**Figure 2:**
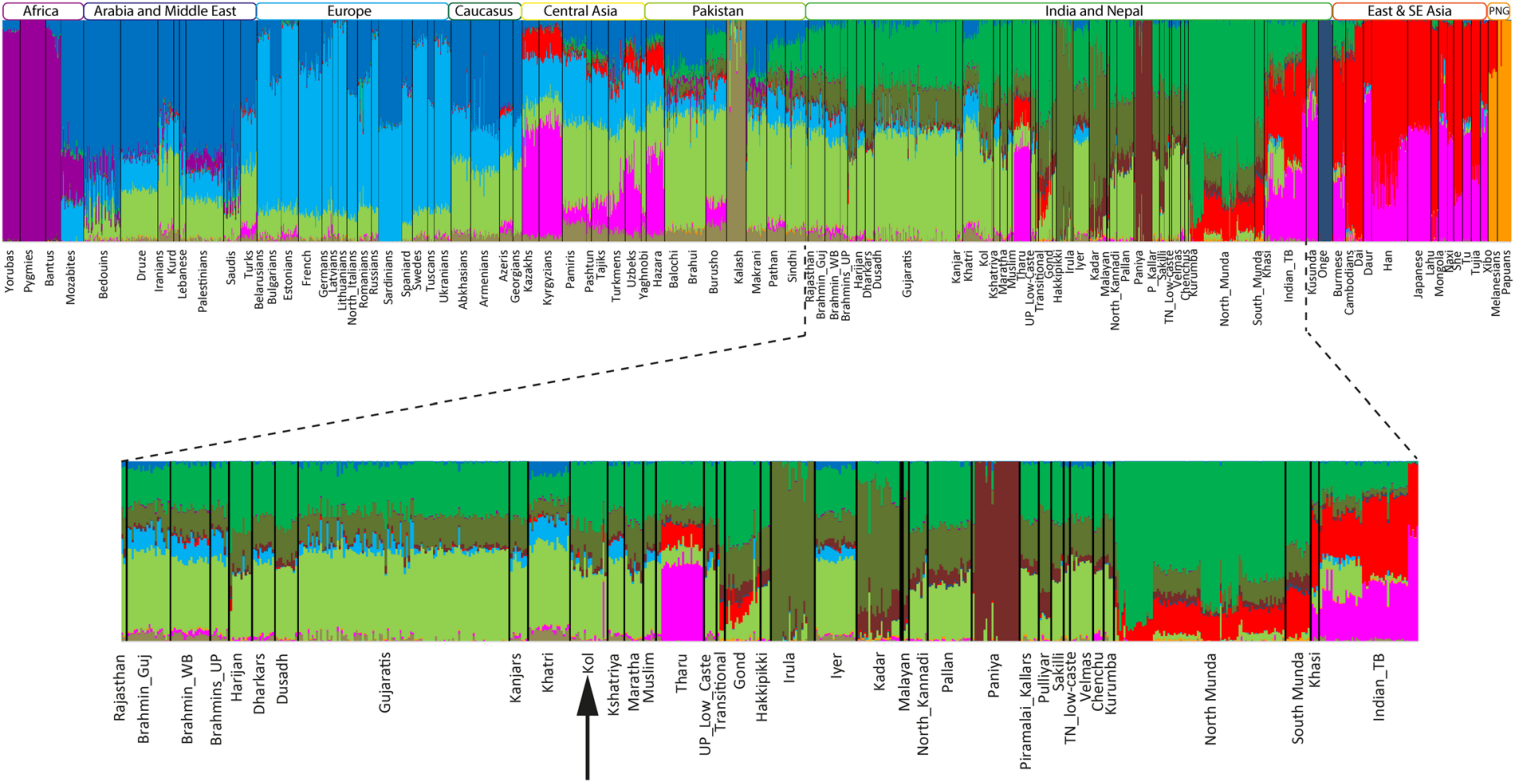
The ADMIXTURE plot at K = 12 showing the ancestry components sharing of Kol population. The full plot of K = 2 to K = 15 has been shown in Supplementary Fig. 1.

In order to understand the genetic component sharing of Kol with the other Indian populations, we have plotted various ancestry components inferred from ADMIXTURE analysis (Supplementary Fig. 1). The log-likelihood estimate was in favour of best K value as K = 12 (Supplementary Fig. 2). Apart from two major components prevalent in South Asia, we also see other minor and population-specific ancestry components (Fig. 2). The majority of these minor components were either sporadic or present among some specific language groups^5,8,25^ e.g. the Southeast/East Asian components among Mundari and Tibeto-Burman speakers^5,25^. However, we also see a South Indian component which was nearly fixed in Irula and is geographically widespread amongst other South Asian populations with a frequency gradient from east to west or south to north (Fig. 2). Amongst both of the Kol groups, all these three components (two major and one minor) were substantially visible. Except for a single sample, none of the Kol individuals showed any East/Southeast Asian specific component significantly (two tailed p value < 0.001), which is otherwise abundant among their geographic and linguistic neighbours (Transitional and Mundari speaking populations). This finding ruled out their recent common ancestry with the Austroasiatic (Mundari) speakers. Hence, together with the PCA, ADMIXTURE analysis also suggested a non-Austroasiatic connection of these ‘Kol’ groups.

We further investigated one outlier sample of Kol which showed high level of East/Southeast Asian ancestry. In the PC analysis, this Kol individual (Kol outlier) aligned along the Trans-Himalayan cline^5^ (Fig. 1B). In terms of population-wise affinity, this individual clustered with the Tharu population of Uttarakhand. In the ADMIXTURE plot (Fig. 2), this individual also showed Tharu like ancestry pattern, confirming the PC analysis result. We retraced our steps from sampling to genotyping of this particular sample, and learnt that the Kol samples were processed in the lab together with the Tharus, and it is likely that one of the ‘Tharu’ sample was mislabelled as ‘Kol’. For further population based analysis (*f*3 and *D* statistics) we omitted this sample from the pool.

For shared drift analysis of Kol groups, we performed the outgroup *f*3 test (Supplementary Fig. 3). The result was consistent with the PCA in terms of their closer affinity with extant South Asian populations (Fig. 1B). Both of the Kol groups showed a significant level of allele sharing with other South Asian populations, particularly with Harijans. Populations who were closer to the Kols in the PCA also showed higher shared drift with the Kols. When we compared the alleles shared with East vs. West Eurasian populations, we observed an inverse affinity of Kol1 vs. Kol2 with the East and West Eurasian populations. Kol1 shared more drift with the West Eurasians, whereas Kol2 shared greater drift with the East Eurasians (Supplementary Fig. 3).

**Figure 3:**
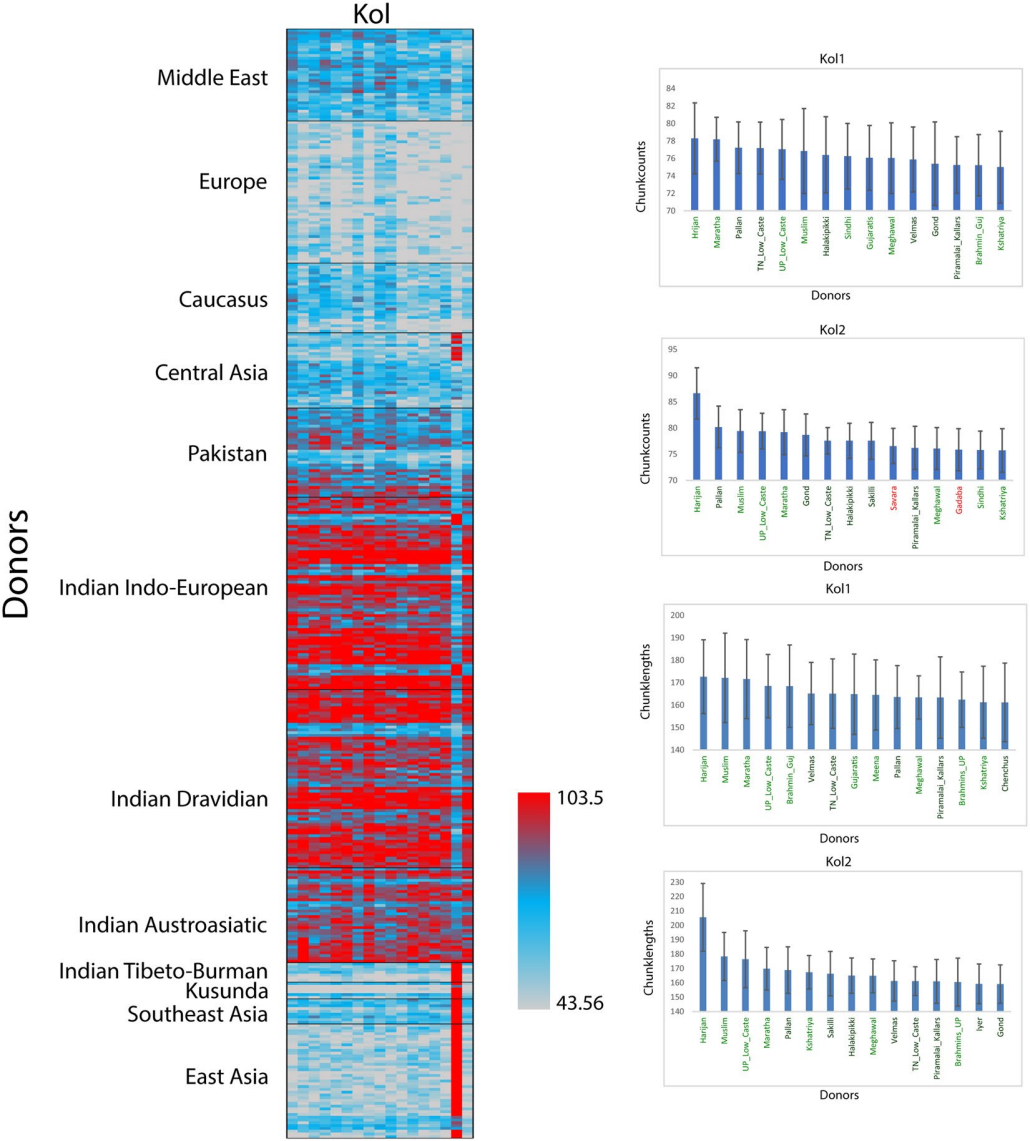
The fineSTRUCTURE analysis showing clustering pattern as well as the chunk sharing of Kol with other Eurasian population. The top 15 donors of chunkcounts and chunklengths for Kol1 and Kol2 were plotted on the right.

In the allele frequency based analysis, the Kols exhibited a closer genetic affinity with the Indo-European scheduled castes and tribal populations, rather than with Austroasiatic or Dravidian populations (Figs. 1B and 2 and Supplementary Figs. 1 and 3). To gain a deeper insight into the extent of genome sharing between the Kols and other South Asian populations, we applied haplotype-based ChromoPainter^26^ and fineSTRUCTURE analysis^26^. On the basis of haplotype sharing amongst the studied groups, we compared the mean chunk counts donated by Eurasian populations with Kol groups (Fig. 3). As expected, Kols received majority of the chunks from South Asian populations when compared with other Eurasians. Amongst the South Asians, the Indo-European scheduled caste population Harijan was the major chunk (chunklength as well as chunkcounts) contributor for both of the Kol groups (Fig. 3). The chunk donation of Austroasiatic (Mundari) populations was significantly lower (two tailed p value < 0.0001). The distinct ancestry of one Kol sample can be also seen in this analysis. The Maximum Likelihood (ML) tree obtained from the fineSTRUCTURE analysis placed both of the Kols together with the Indo-European populations (Supplementary Fig. 4). Kol1 and Kol2 fell in to two distinct clusters. Together with other populations, Kol1 is distributed in to two sub-clusters, whereas Kol2 form their five largely own sub-clusters, where one was shared with the Harijans (Supplementary Fig. 4).

To see, if the Kol1 and Kol2 belong to same pan-Kol ancestry, we computed *D* statistics asking if there is any population which share more alleles with either of these (Table 1). When we filtered the top 10 *D* values for Kol populations, we didn’t find any population which shared significantly more alleles than Kol1 shares with Kol2. Thus both of the Kol groups share a more recent common ancestry. To investigate further the inbreeding and relatedness among both the Kol groups, we analysed Runs of Homozygosity (RoH) in the populations^27–29^ (Supplementary Fig. 5). In an inbred populations RoH tend to be longer and recent in time as recombination doesn’t get enough time to break the identical-by-descent segments. Conversely, shorter RoH segments are considered to be older. Both of the Kol groups showed lower RoH segments when compared with the Austroasiatic (Mundari) speaking populations, suggesting their different population history as well as high effective population size (Ne).

**Table 1:** The top ten values of *D* statistics showing the gene flow between Kol and other Indian populations.

Gp1	Gp2	Gp3	*D* value	*Z* score
Kol1	Kol2	Kurmi	−0.001	−0.49
Kol1	Kol2	Harijan	−0.0017	−1.729
Kol1	Kol2	UP_Low_Caste	−0.0017	−1.369
Kol1	Kol2	Muslim	−0.0025	−2.021
Kol1	Kol2	Dusadh	−0.0039	−3.519
Kol1	Kol2	Gujaratis	−0.0043	−5.138
Kol1	Kol2	Chenchus	−0.0043	−3.289
Kol1	Kol2	Kanjars	−0.0048	−4.16
Kol1	Kol2	North_Kannadi	−0.0049	−4.343
Kol1	Kol2	Sakilli	−0.005	−3.768

D = (Yoruba, Gp1; Gp2,Gp3).

In order to gain information about their maternal ancestry sharing, we analysed mitochondrial DNA (mtDNA) sequences of the HVS-I (hypervariable segment I) and selected coding regions. Both of the Kol groups shared M2, M3, M18, M30 and R5 haplogroups (Supplementary Fig. 6 and Supplementary Table 2). Our previous study has identified haplogroup R7 as highly frequent haplogroup among North Mundari speakers^30^. However, we didn’t find any sample of Kol belonging to haplogroup R7 (Supplementary Table 2). The mtDNA haplogroups of Kols were quite distinct from the general trend of Mundari populations^10,22,30,31^. We utilised haplogroup frequencies to calculate the principal components. We have used geographic labels in one plot and linguistic labels in another plot (Supplementary Fig. 7). In the geographical placement, the pattern followed the isolation-by-distance model. The Uttar Pradesh/Madhya Pradesh Kol (Kol1) clustered with Uttar Pradesh and Madhya Pradesh populations, whereas Maharashtra Kol (Kol2) clustered with the neighbouring Andhra Pradesh populations (Supplementary Fig. 7a). In terms of linguistic affiliation, Kol1 clustered closely with populations speaking Indo-European languages, whereas Kol2 cluster with Andhra Pradesh Dravidian speakers (Supplementary Fig. 7b). Therefore, their maternal ancestry also precludes their Austroasiatic (Munda) affinity. Yet, previous studies have identified the Austroasiatic language communities of South Asia as the result of a gender biased linguistic intrusion, with resulted from the spread of the language by male speakers who introduced the predominant Munda paternal lineage along with a small but recognisable Southeast Asian autosomal component^26^. However, because of the absence of Y chromosomal haplogroup information from the Kol groups, we are unable to test their paternal affiliation.

In conclusion, contrary to what is suggested by their name, we found no recent common genetic ancestry of these two Kol groups with the Austroasiatic (Mundari) speakers. The genetic structure of these Kols is more akin to the North Indian Indo-European scheduled caste population known as the Harijan. This finding matches our recent finding that Harijans and Kols shared short IBD (identical by descent) segments with Indian Mundari speakers^25^ rejecting any recent geneflow or common ancestry. Our analysis also discards a case of recent language shift, as none of the Kol carried the signal of Southeast Asian ancestry that is present in Austroasiatic (Mundari) populations.

Thus, our detailed analysis on one of the major South Asian tribal populations, support a deeply rooted endogamy, which not only exist among caste populations, but also present among tribal populations. Particularly in this case, our sampled Kols lived side-by-side with the Mundari populations. Our finding leaves us with the question as to whether the sampled ‘Kol’ populations could represent the remnant of ancestral Kol before the ancestors of Munda were linguistically assimilated by incursive Austroasiatic speakers. Since antiquity and even in modern times, in the social climbing process, entire ethnic groups and language communities have been known to pass themselves off as another caste or linguistic group that happens to rank higher in the caste hierarchy^26^. The present study presents what appears to be the first genetic evidence for such a collective ethnolinguistic identity reassignment.

## Materials and Methods

To sample Kol population, in the first phase, we surveyed 566 individuals from 12 villages covering three major states of their settlement (Uttar Pradesh, Madhya Pradesh and Maharashtra). It was striking that, in our survey to the sampling regions (Fig. 1A), we did not find a single Kol individual, speaking or having knowledge of Mundari languages. All of the individuals surveyed were fluent in the local Indo-Aryan languages instead, i.e. Bhojpuri-Bagheli in Uttar Pradesh and Madhya Pradesh, Marathi in Maharashtra. Since all early anthropological and linguistic studies on Kols unanimously established their linguistic affinity as speaking Ho or other languages of the Kherwarian cluster within the Northern Branch of the Munda subgroup within Austroasiatic^7–9^, in the case of the linguistically assimilated young Kols whom we sampled, we double-checked their ethnolinguistic identity with linguistic expert involved in the study. Since language shift has previously been reported amongst Central Indian tribes^10,32^, we presume that this is also the case with the Kols sampled in the present study. However, we note that a similar model did not appear to apply to the Gond in our previous studies^7,20^. Therefore, in this study we used large number of autosomal and mitochondrial DNA markers to investigate the conflicting association of Kols as well as their inter and intra population affinities (Supplementary Tables 1 and 2).

We finally collected blood samples of the Kol population from 55 unrelated individuals with informed consent. We avoided people related up to three generations. The first group of Kol (Kol1) was sampled from the geographic borders of Uttar Pradesh and Madhya Pradesh states and second group (Kol2) was collected from Maharashtra state (Fig. 1A). Both of these sampling points were from the places where the Kol are highly concentrated. The DNA was isolated and quantified from standard protocol^33^. We further selected 17 high-quality samples (seven Kol1 and ten Kol2) and generated Illumina 650 K genotype data. This data was released in our earlier publication^23^. All the 55 samples were sequenced for the mtDNA HVS-I region (Supplementary Table 2). We first classified them in their tentative haplogroups, based on the HVS-I mutation and further confirmed these findings by genotyping for coding region mutations (Supplementary Table 2). This study was approved by the ethical committee of the Banaras Hindu University, Varanasi, India. All methods were performed in accordance with the relevant guidelines and regulations.

For autosomal data we used PLINK1.9^34^ for quality control and data management. We merged the data of the 17 Kol samples with the 1756 samples belonging to 119 world populations (Supplementary Table 1). Similar to our previous studies, SNPs with more than 3% missingness across individuals or with a minor allele frequency less than 10% were removed^23,35^. We have also removed SNPs deviating from Hardy-Weinberg equilibrium^36^. After all quality control measures, we obtained 258311 high quality SNPs, which we used for all our analyses. We classified Indian populations according to their language group. For the populations having conflicted linguistic affiliation, we followed Kumar and Reddy^32^ and classified them as ‘Transitional’. To remove background linkage disequilibrium (LD) that can affect both principal component analysis (PCA) and ADMIXTURE, we thinned the data set by removing one SNP of any pair in strong LD r2 > 0.4, in a window of 200 SNPs (sliding the window by 25 SNPs at a time). We performed PC analysis using the smartpca programme of the EIGENSOFT package^37^ with the default settings to capture genetic variability described by the first ten components. We ran unsupervised ADMIXTURE v1.3^38^ with a random seed number generator on the LD-pruned data set 25 times from K = 2 to K = 15. The best supported clustering was shown at K = 12^21,23^. Given the result of the PC and ADMIXTURE analysis, we removed one outlier sample from the Kol2 group for further population-based analysis. The outgroup *f*3 statistics^39^ were calculated as *f*3 = (Kol1/Kol2, X; Yoruba), where X was any other population and Yoruba served as an outgroup. To investigate the pan-Kol ancestry, we performed *D* statistics by taking African Yoruba as an outlier *D* = (Yoruba, Kol1; Kol2, X), whereby X was the other Indian populations. For haplotype-based comparison ChromoPainter v1^26^ and fineSTRUCTURE v1^26^ were used to perform an MCMC iteration, using 10 M burning runtime and the same MCMC iterations. We first phased our samples with Beagle 3.3.2^40^ and modelled haplotype sharing among studied individuals by using ChromoPainter. The ChromoPainter creates a co-ancestry matrix where each and every individual share chunkcounts and chunklength with each other^26^. Thereafter, fineSTRCUTURE algorithm cluster them in to subgroups based on the pattern of co-ancestry matrix. The data of fineSTRUCTURE were used to construct the maximum likelihood (ML) tree using MEGA^41^. Runs of homozygosity (RoH) were performed to investigate the inbreeding and ancestral homozygous component sharing. For RoH estimation, we applied window size 1,000 kb, a minimum of 100 SNPs per window allowing one heterozygous and five missing calls per window^27^.

## Supplementary information

Supplementary Information.
