# Supplementary material for: Genetic and linguistic non-correspondence suggests evidence for collective social climbing in the Kol tribe of South Asia
Source: Sci Rep. 2020 Mar 27;10:5593. doi: 10.1038/s41598-020-61941-z (PMC7101412; doi:10.1038/s41598-020-61941-z)
Supplement: Supplementary file 1 — Supplementary Information. [file 41598_2020_61941_MOESM1_ESM.pdf]

Supplementary file

Genetic and linguistic non-correspondence suggests evidence for collective social climbing in the Kol tribe of South Asia

Anshika Srivastava[1¶], Prajjval Pratap Singh[1¶], Audditiya Bandopadhyay[1¶], Pooja Singh[1], Debashruti Das[1], Rakesh Tamang[2], Akhilesh Kumar Chaubey[3], Pankaj Shrivastava[4], George van Driem[5,6,γ] & Gyaneshwer Chaubey[1,7*γ]

[1]Cytogenetics Laboratory, Department of Zoology, Banaras Hindu University, Varanasi, 221005, India
[2]Department of Zoology, University of Calcutta, Kolkata 700019, India
[3]Krishi Vigyan Kendra, Singrauli, Jawaharlal Nehru Krishi Vishwavidyalay, Jabalpur, Madhya Pradesh 462038, India.
[4]DNA Fingerprinting Unit, State Forensic Science Laboratory, Department of Home (Police), Government of MP, Sagar 470001, India
[5]Institut für Sprachwissenschaft, Universität Bern, 3012 Bern, Switzerland
[6]Sydney Social Sciences and Humanities Advanced Research Centre, University of Sydney, Australia
[7] Estonian Biocentre, Institute of Genomics, University of Tartu, Tartu, 5100, Estonia

[¶] Equal contributions

[γ] Equal Senior authors

Supplementary Figure 1. The ADMIXTURE plot of K=2 to K=15 of all the studied populations.

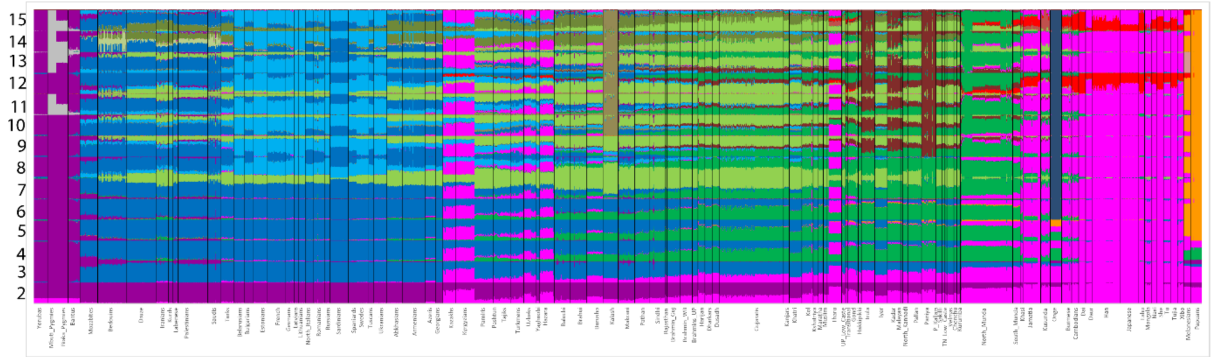

Supplementary Figure 2. The plot of loglikelihood values obtained from running the ADMIXTURE analysis.

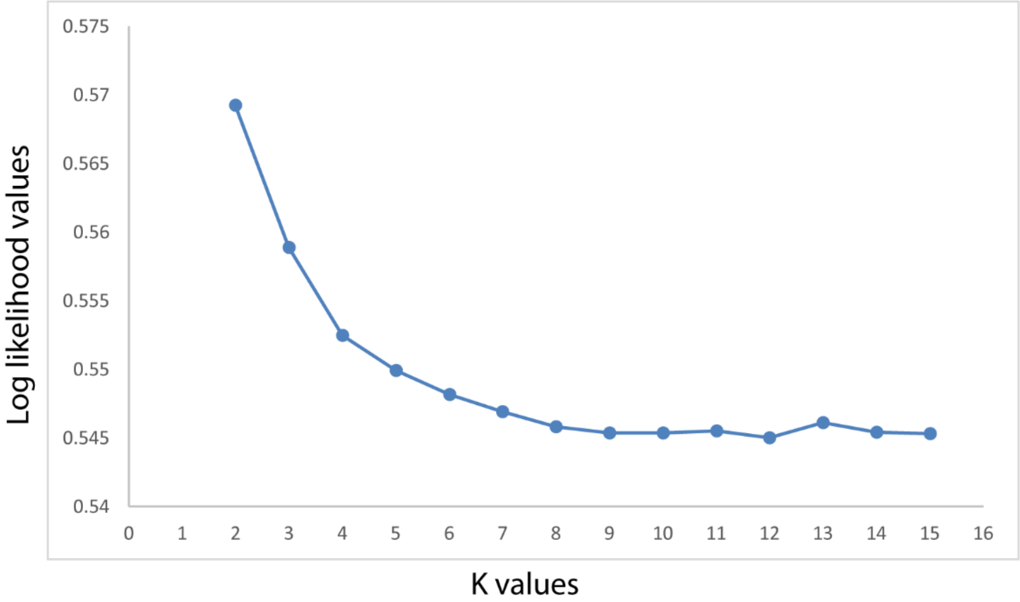

Supplementary Figure 3. The outgroup *f*3 statistics of the Kol1 and Kol2 populations showing the shared drift with the Eurasian samples.

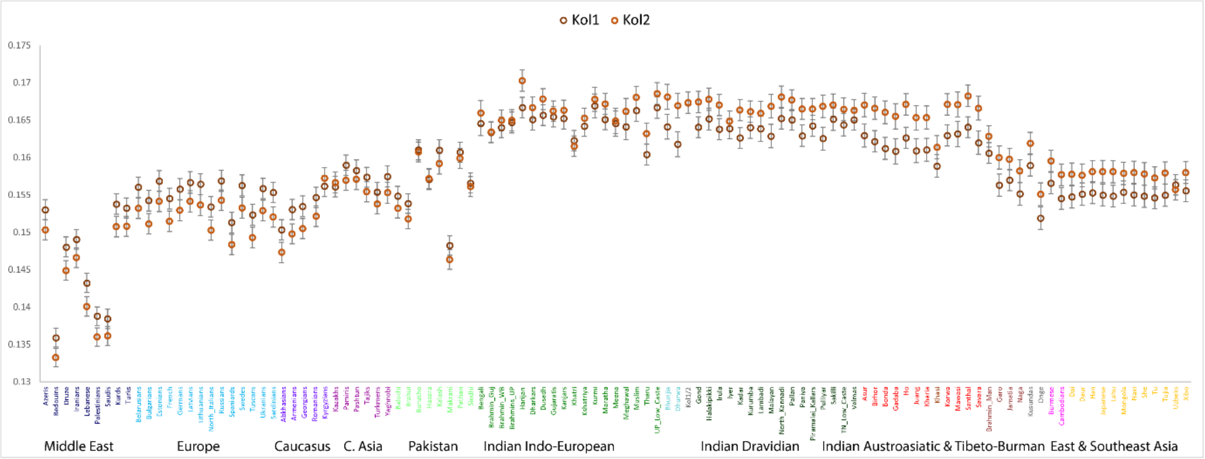

Supplementary Figure 4. The Maximum Likelihood (ML) tree obtained from fineSTRUCTURE analysis showing the placement of the Kol populations. The most recent clusters of Kol1 and Kol2 have been zoomed-in.

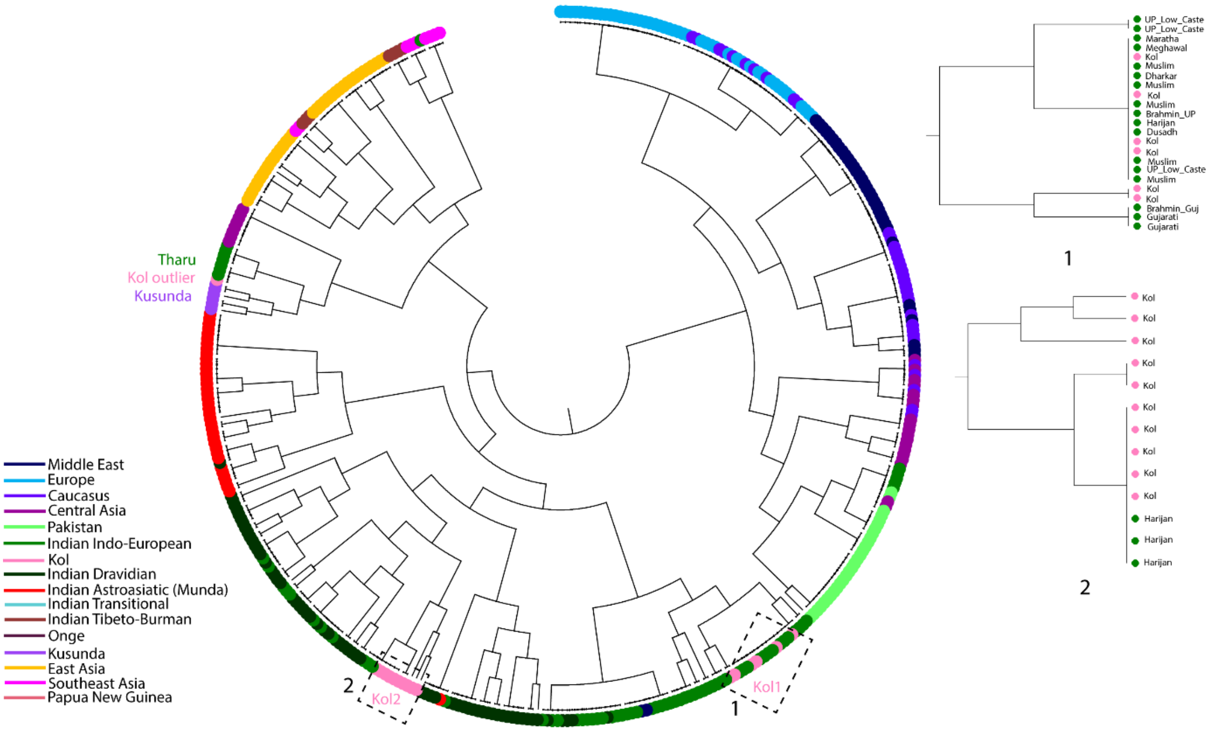

Supplementary Figure 5. The plot of Runs of Homozygosity (RoH) of Kol populations.

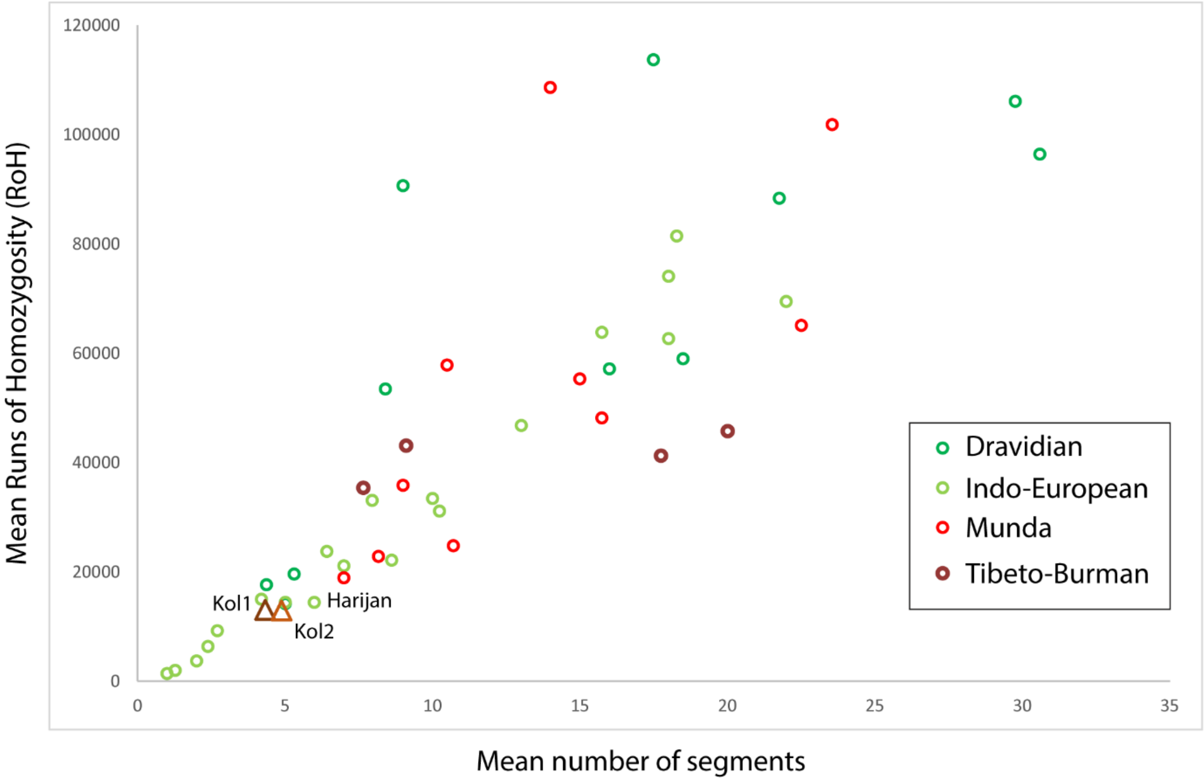

Supplementary Figure 6. The Venn diagram showing the maternal haplogroups shared as well as unique for both of the Kol groups.

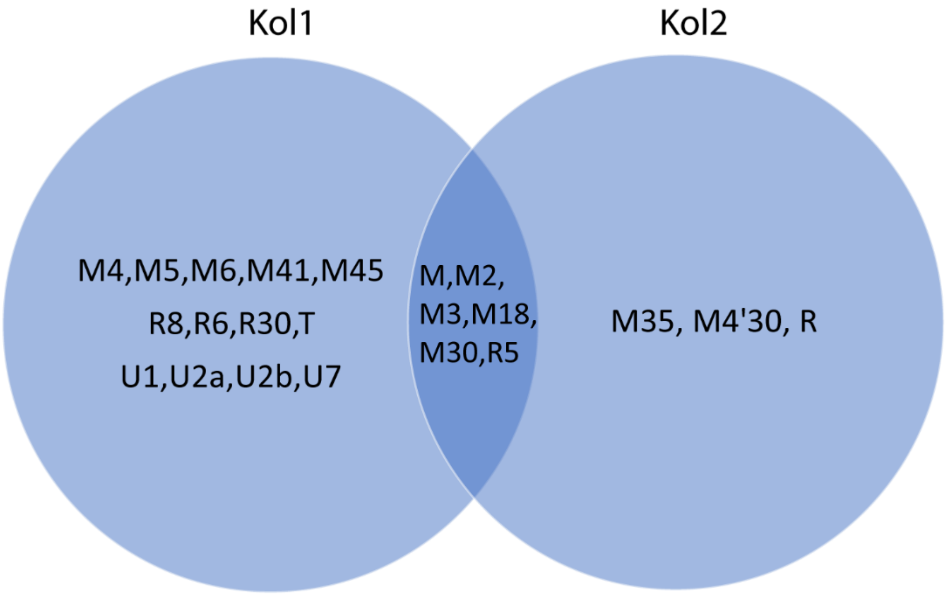

Supplementary Figure 7. The principal component analysis (PCA) based on mitochondrial DNA haplogroup frequency. **a)** PCA plot based on geography **b)** PCA plot based on linguistic affiliation.

Supplementary file

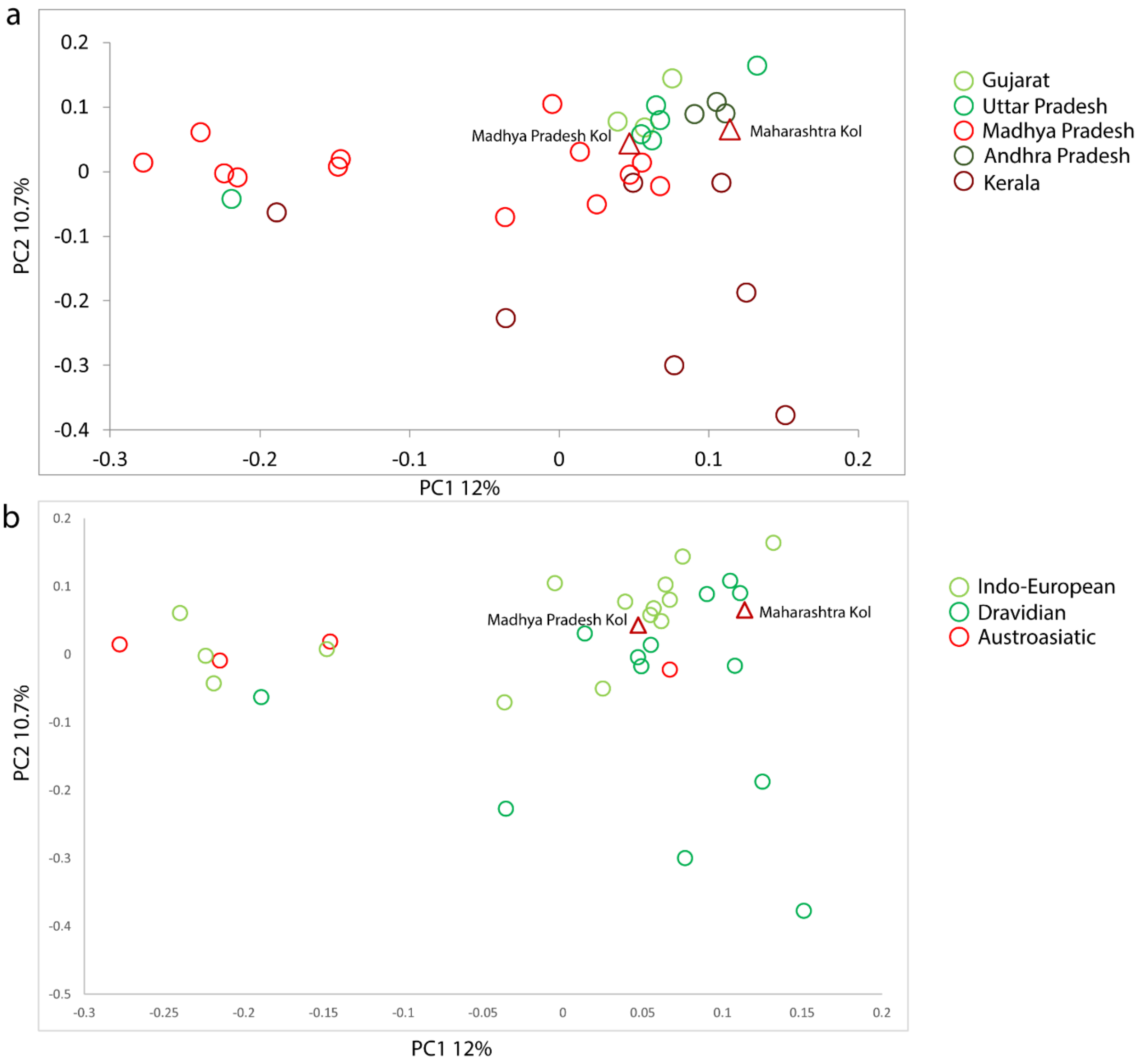

Supplementary Table 1. The details of studied populations for high density autosomal SNPs included in the present study

Region	Population	n	*f3/f4/D*	ADMIXTURE	PCA	fineSTRUCTURE	References
Africa	Bantus	18	18	18	-	18	Li et al. 2008
	Mbuti_Pygmies	13	13	13	-	13	Li et al. 2008
	Biaka_Pygmies	18	18	18	-	18	Li et al. 2008
	Yorubas	21	21	21	-	21	Li et al. 2008
	Mozabites	27	27	27	-	27	Li et al. 2008
Middle East	Saudis	20	20	20	20	20	Behar et al. 2010, Atzmon et al. 2010
	Bedouins	45	45	45	45	45	Li et al. 2008
	Druze	45	45	45	45	45	Li et al. 2008
	Palestinians	46	46	46	46	46	Li et al. 2008
	Turks	19	19	19	19	19	Behar et al. 2010
	Lebanese	7	7	7	7	7	Behar et al. 2010, Atzmon et al. 2010
	Kurds	6	6	6	6	6	Behar et al. 2010
	Iranians	19	19	19	19	19	Behar et al. 2013

Supplementary file

Europe	French	28	28	28	28	28	Li et al. 2008
	Latvians	6	6	6	6	6	Kushniarevich et al. 2015
	Lithunians	10	10	10	10	10	Behar et al. 2010
	Spaniards	12	12	12	12	12	Behar et al. 2010
	Germans	12	12	12	12	12	Yunusbayev et al. 2015
	Estonians	21	21	21	21	21	Raghavan et al. 2014, Kushniarevich et al. 2015
	Belarusians	16	16	16	16	16	Behar et al. 2010, Behar et al. 2013
	Sardinians	28	28	28	28	28	Li et al. 2008
	Swedes	18	18	18	18	18	Behar et al. 2010
	North_Italians	12	12	12	12	12	Li et al. 2008
	Tuscans	7	7	7	7	7	Li et al. 2008
	Ukranians	20	20	20	20	20	Yunusbayev et al. 2011
	Russians	8	8	8	8	8	Li et al. 2008
Caucasus	Armenians	35	35	35	35	35	Behar et al. 2010, Yunusbayev et al. 2011
	Romanians	16	16	16	16	16	Yunusbayev et al. 2011
	Georgians	10	10	10	10	10	Behar et al. 2010, Yunusbayev et al. 2011
	Abhkasians	23	23	23	23	23	Yunusbayev et al. 2011
	Azeris	16	16	16	16	16	Yunusbayev et al. 2015
	Bulgarians	13	13	13	13	13	Yunusbayev et al. 2011
Central Asia	Tajiks	20	20	20	20	20	Yunusbayev et al. 2011
	Pamiris	29	29	29	29	29	Yunusbayev et al. 2015
	Yaghnobi	5	5	5	5	5	Yunusbayev et al. 2015
	Turkmens	20	20	20	20	20	Yunusbayev et al. 2011
	Krygzians	28	28	28	28	28	Raghavan et al. 2014
	Uzbeks	19	19	19	19	19	Behar et al. 2010
	Kazakhs	20	20	20	20	20	Raghavan et al. 2014
South Asia	Kharia	2	2	2	2	2	Chaubey et al. 2011
	Juang	2	2	2	2	2	Chaubey et al. 2011
	Meena	1	1	1	1	1	Metspalu et al. 2011
	Kanjars	8	8	8	8	8	Metspalu et al. 2011
	Malayan	2	2	2	2	2	Behar et al. 2010
	Bengali	1	1	1	1	1	Metspalu et al. 2011
	Birhor	16	16	16	16	16	Moorjani et al 2013
	Gadaba	1	1	1	1	1	Chaubey et al. 2011
	Savara	2	2	2	2	2	Chaubey et al. 2011
	Pashtun	5	5	5	5	5	DiCristofaro et al. 2013
	Piramalai_Kallars	8	8	8	8	8	Metspalu et al. 2011
	Kol	17	17	17	17	17	Metspalu et al. 2011
	Pallan	20	20	20	20	20	Basu et al 2016
	Sakilli	4	4	4	4	4	Behar et al. 2010
	Pulliyar	5	5	5	5	5	Metspalu et al. 2011
	Paniya	22	22	22	22	22	Behar et al. 2010
	Bhujia	1	1	1	1	1	Metspalu et al. 2011
	Dharkars	11	11	11	11	11	Metspalu et al. 2011
	Kurmi	1	1	1	1	1	Metspalu et al. 2011
	Brahmin_Guj	20	20	20	20	20	Basu et al 2016
	Kurumba	4	4	4	4	4	Reich et al. 2009, Metspalu et al. 2011

	Kadar	20	20	20	20	20	Basu et al 2016
	Brahmin_WB	18	18	18	18	18	Basu et al 2016
	North Kannadi	8	8	8	8	8	Behar et al. 2010
	Brahui	25	25	25	25	25	Li et al. 2008
	Balochi	24	24	24	24	24	Li et al. 2008
	Hazara	22	22	22	22	22	Li et al. 2008
	Makrani	25	25	25	25	25	Li et al. 2008
	Sindhi	24	24	24	24	24	Li et al. 2008
	Pathan	22	22	22	22	22	Li et al. 2008
	Kalash	23	23	23	23	23	Li et al. 2008
	Burusho	25	25	25	25	25	Li et al. 2008
	Mawasi	1	1	1	1	1	Chaubey et al. 2011
	Brahmin_Man	20	20	20	20	20	Basu et al 2016
	Maratha	7	7	7	7	7	Basu et al 2016
	Muslim	5	5	5	5	5	Metspalu et al. 2011
	Gujaratis	100	100	100	100	100	HapMap 3
	Brahmins_UP	8	8	8	8	8	Metspalu et al. 2011
	Kshatriya	7	7	7	6	7	Metspalu et al. 2011
	Asur	2	2	2	2	2	Chaubey et al. 2011
	Lambadi	1	1	1	1	1	Metspalu et al. 2011
	Velmas	10	10	10	10	10	Reich et al. 2009, Metspalu et al. 2011
	Tharu	22	22	22	22	22	Reich et al. 2009, Metspalu et al. 2011
	Meghwal	1	1	1	1	1	Reich et al. 2009
	Santhal	21	21	21	21	21	Reich et al. 2009, Chaubey et al. 2011
	Gond	16	16	16	16	16	Metspalu et al. 2011
	Harijan	10	10	10	10	10	Metspalu et al. 2011
	Bonda	4	4	4	4	4	Chaubey et al. 2011
	Dusadh	10	10	10	10	10	Metspalu et al. 2011
	Halakipikki	4	4	4	4	4	Metspalu et al. 2011
	Irula	20	20	20	20	20	Moorjani et al 2013
	Iyer	19	19	19	19	19	Basu et al 2016
	Khatri	19	19	19	19	19	Basu et al 2016
	Korwa	18	18	18	18	18	Basu et al 2016
	Dhruwa	1	1	1	1	1	Metspalu et al. 2011
	UP_Low_Caste	5	5	5	5	5	Metspalu et al. 2011
	TN_Low caste	2	2	2	2	2	Metspalu et al. 2011
	Chenchus	4	4	4	4	4	Metspalu et al. 2011
	Ho	23	23	23	23	23	Basu et al 2016
Himalayan and Adjoining Populations (HAAP)	Khasi	3	3	3	3	3	Chaubey et al. 2011
	Kusunda	14	14	14	14	14	Rasmussen et al. 2011
	Garo	4	4	4	4	4	Chaubey et al 2010
	Naga	5	5	5	5	5	Metspalu et al 2011
	Jamatia	18	18	18	18	18	Basu et al 2016
Andamanese	Onge	17	17	17	17	17	Basu et al 2016
East and South East Asia	Burmese	15	15	15	15	15	Chaubey et al. 2011
	Cambodians	10	10	10	10	10	Li et al. 2008
	Dai	10	10	10	10	10	Li et al. 2008

	Lahu	8	8	8	8	8	Li et al. 2008
	Naxi	7	7	7	7	7	Li et al. 2008
	She	10	10	10	10	10	Li et al. 2008
	Han	44	44	44	44	44	Li et al. 2008
	Tujia	10	10	10	10	10	Li et al. 2008
	Tu	10	10	10	10	10	Li et al. 2008
	Xibo	9	9	9	9	9	Li et al. 2008
	Daur	9	9	9	9	9	Li et al. 2008
	Mongola	10	10	10	10	10	Li et al. 2008
	Japanese	28	28	28	28	28	Li et al. 2008
PNG	Melanesians	10	10	10	10	10	Li et al. 2008
	Papuans	17	17	17	17	17	Li et al. 2008
	Total samples	1773	1773	1773	1675	1773	

References

Atzmon, G. et al. Abraham's children in the genome era: major Jewish diaspora populations comprise distinct genetic clusters with shared Middle Eastern Ancestry. Am J Hum Genet. 2011; 86: 850–859.

Basu, A., Sarkar-Roy, N. & Majumder, P.P. Genomic reconstruction of the history of extant populations of India reveals five distinct ancestral components and a complex structure. Proc. Natl. Acad. Sci. USA 2016; 113: 1594–1599.

Behar DM, Yunusbayev B, Metspalu M et al: The genome-wide structure of the Jewish people. Nature 2010; 466: 238–242.

Chaubey G, Metspalu M, Choi Y et al: Population genetic structure in Indian Austroasiatic speakers: the role of landscape barriers and sex-specific admixture. Mol Biol Evol 2011; 28: 1013–1024.

DiCristofaro J, et al. Afghan Hindu Kush: where Eurasian sub-continent gene flows converge. PLoS One. 2013;8:e76748. doi: 10.1371/journal.pone.0076748.

International HapMap 3 Consortium International HapMap 3 Consortium, Altshuler DM International HapMap 3 Consortium, Gibbs RA et al. Integrating common and rare genetic variation in diverse human populations. Nature 2010; 467: 52–58.

Kushniarevich A, et al. Genetic heritage of the balto-slavic speaking populations: A synthesis of autosomal, mitochondrial and Y-chromosomal data. PLoS One. 2015;10:e0135820.

Li JZ, Absher DM, Tang H et al: Worldwide human relationships inferred from genome-wide patterns of variation. Science 2008; 319: 1100–1104.

Metspalu M, Romero IG, Yunusbayev B et al: Shared and unique components of human population structure and genome-wide signals of positive selection in South Asia. Am J Hum Genet 2011; 89: 731–744

Moorjani P, Thangaraj K, Patterson N et al: Genetic evidence for recent population mixture in India. Am J Hum Genet 2013; 93: 422–438.

Raghavan M, Skoglund P, Graf KE et al: Upper Palaeolithic Siberian genome reveals dual ancestry of Native Americans. Nature 2014; 505: 87–91.

Rasmussen M, Guo X, Wang Y et al: An Aboriginal Australian genome reveals separate human dispersals into Asia. Science 2011; 334: 94–98.

Reich D, Thangaraj K, Patterson N, Price AL, Singh L: Reconstructing Indian population history. Nature 2009; 461: 489–494.

Yunusbayev B, Metspalu M, Metspalu E et al: The genetic legacy of the expansion of Turkic-speaking nomads across Eurasia. PLoS Genet 2015; 11: e1005068.

Supplementary Table 2. The mtDNA HVS-I and coding region mutations of Kol population

State	Language	Population	Id	HVS-I (-16000nt)	Coding region	haplogroup
Uttar Pradesh/Madhya Pradesh	Indo-European	Kol1	289	129-223-276-289-342	489-1888-10400	**M5**
Uttar Pradesh/Madhya Pradesh	Indo-European	Kol1	290	129-145-223-261-311	489-10400-12007	**M4a**

Supplementary file

Uttar Pradesh/Madhya Pradesh	Indo-European	Kol1	291	145-223-266-355	489-10400	**M**
Uttar Pradesh/Madhya Pradesh	Indo-European	Kol1	292	223-234	489-10400	**M**
Uttar Pradesh/Madhya Pradesh	Indo-European	Kol1	294	51-206C-230-311	1811-12308	**U2a**
Uttar Pradesh/Madhya Pradesh	Indo-European	Kol1	295	189-234-240-247	489-10400	**M**
Uttar Pradesh/Madhya Pradesh	Indo-European	Kol1	296	292	9449-15326	**R8**
Uttar Pradesh/Madhya Pradesh	Indo-European	Kol1	297	309-318C	1811-12308	**U7**
Uttar Pradesh/Madhya Pradesh	Indo-European	Kol1	298	223-311	489-10400-12007	**M4**
Uttar Pradesh/Madhya Pradesh	Indo-European	Kol1	299	129-223-291	489-1888-10400	**M5**
Uttar Pradesh/Madhya Pradesh	Indo-European	Kol1	300	126-223-287G-300-309	489-10400	**M**
Uttar Pradesh/Madhya Pradesh	Indo-European	Kol1	302	129-172-223	489-1888-10400	**M5**
Uttar Pradesh/Madhya Pradesh	Indo-European	Kol1	303	223-300	489-9180-10400	**M45**
Uttar Pradesh/Madhya Pradesh	Indo-European	Kol1	304	51-223-231-311-362	461-489-10400	**M6**
Uttar Pradesh/Madhya Pradesh	Indo-European	Kol1	305	126-181-209-256	8584-15326	**R30**
Uttar Pradesh/Madhya Pradesh	Indo-European	Kol1	306	51-93-206C-230-311	1811-12308	**U2a**
Uttar Pradesh/Madhya Pradesh	Indo-European	Kol1	307	51-206C-230-304-311	1811-12308	**U2a**
Uttar Pradesh/Madhya Pradesh	Indo-European	Kol1	309	126-294-296-325	4216-15326	**T**
Uttar Pradesh/Madhya Pradesh	Indo-European	Kol1	310	266-304	8594-15326	**R5**
Uttar Pradesh/Madhya Pradesh	Indo-European	Kol1	311	129-183C-223	489-1888-10400	**M5**
Uttar Pradesh/Madhya Pradesh	Indo-European	Kol1	312	126-223-319	482-489-10400	**M3**
Uttar Pradesh/Madhya Pradesh	Indo-European	Kol1	313	189-223-274	447G-489-10400	**M2**
Uttar Pradesh/Madhya Pradesh	Indo-European	Kol1	314	189-249-274	2218-12308	**U1a**
Uttar Pradesh/Madhya Pradesh	Indo-European	Kol1	319	266-304	8594-15326	**R5**
Uttar Pradesh/Madhya Pradesh	Indo-European	Kol1	320	223-261-278	489-10400	**M**
Uttar Pradesh/Madhya Pradesh	Indo-European	Kol1	321	189-304-311	8594-15326	**R5**
Uttar Pradesh/Madhya Pradesh	Indo-European	Kol1	322	185-189-223-289-362	489-10400	**M**
Uttar Pradesh/Madhya Pradesh	Indo-European	Kol1	323	223	489-10400	**M**
Uttar Pradesh/Madhya Pradesh	Indo-European	Kol1	326	223-234-305-311-362	489-10400-15431	**M30**
Uttar Pradesh/Madhya Pradesh	Indo-European	Kol1	327	227-245-266-278-362	12285-15326	**R6**
Uttar Pradesh/Madhya Pradesh	Indo-European	Kol1	328	223-327	375-489-10400	**M41**
Uttar Pradesh/Madhya Pradesh	Indo-European	Kol1	329	51-209-239-352-353	1811-12308	**U2b**
Uttar Pradesh/Madhya Pradesh	Indo-European	Kol1	330	182c-183C-189-304-311	8594-15326	**R5**
Uttar Pradesh/Madhya Pradesh	Indo-European	Kol1	331	223	489-10400	**M**
Uttar Pradesh/Madhya Pradesh	Indo-European	Kol1	332	206C-230-311-364	1811-12308	**U2a**
Uttar Pradesh/Madhya Pradesh	Indo-European	Kol1	333	189-222-298-299	8584-15326	**R30**
Uttar Pradesh/Madhya Pradesh	Indo-European	Kol1	334	51-168-192-243-287	1811-12308	**U2b**
Uttar Pradesh/Madhya Pradesh	Indo-European	Kol1	335	223-318C-364	489-10400-12007	**M18**
Maharashtra	Indo-European	Kol2	D361	223-304	489-10400-12561	**M35**
Maharashtra	Indo-European	Kol2	D363	223	489-10400-15431	**M30c**
Maharashtra	Indo-European	Kol2	D364	126-181-209	15326	**R**
Maharashtra	Indo-European	Kol2	D366	223-519	489-10400	**M**
Maharashtra	Indo-European	Kol2	D367	223	489-10400	**M**
Maharashtra	Indo-European	Kol2	D368	104-223-234-243-244-519	489-10400	**M**
Maharashtra	Indo-European	Kol2	D369	15885-223	489-10400	**M**
Maharashtra	Indo-European	Kol2	D370	15888-223-318T-325-519	489-10400-12007	**M18**
Maharashtra	Indo-European	Kol2	D371	15885-223	489-10400	**M**
Maharashtra	Indo-European	Kol2	D372	126-180-223	482-489-10400	**M3**
Maharashtra	Indo-European	Kol2	D373	093-266-304-519-524	8594-15326	**R5**
Maharashtra	Indo-European	Kol2	D375	223-318T-325-519	489-10400-12007	**M18**

Supplementary file

Maharashtra	Indo-European	Kol2	D380	266-304-309-325-519-527	8594-15326	**R5**
Maharashtra	Indo-European	Kol2	D381	304-519	8594-15326	**R5**
Maharashtra	Indo-European	Kol2	D382	126-189-223	482-489-10400	**M3**
Maharashtra	Indo-European	Kol2	D383	189-223	489-10400-12007	**M4'30**
Maharashtra	Indo-European	Kol2	D384	223-274-311-319	447G-489-10400	**M2**
